# Hypertrophy and ER Stress Induced by Palmitate Are Counteracted by Mango Peel and Seed Extracts in 3T3-L1 Adipocytes

**DOI:** 10.3390/ijms24065419

**Published:** 2023-03-12

**Authors:** Giovanni Pratelli, Diana Di Liberto, Daniela Carlisi, Sonia Emanuele, Michela Giuliano, Antonietta Notaro, Anna De Blasio, Giuseppe Calvaruso, Antonella D’Anneo, Marianna Lauricella

**Affiliations:** 1 Section of Biochemistry, Department of Biomedicine, Neurosciences and Advanced Diagnostics (BIND), University of Palermo, 90127 Palermo, Italy; 2 Laboratory of Biochemistry, Department of Biological, Chemical and Pharmaceutical Sciences and Technologies (STEBICEF), University of Palermo, 90127 Palermo, Italy

**Keywords:** mango peel extracts, mango seed extracts, saturated fatty acids, 3T3-L1 adipocytes, ER stress, AMPK, Nrf2

## Abstract

A diet rich in saturated fatty acids (FAs) has been correlated with metabolic dysfunction and ROS increase in the adipose tissue of obese subjects. Thus, reducing hypertrophy and oxidative stress in adipose tissue can represent a strategy to counteract obesity and obesity-related diseases. In this context, the present study showed how the peel and seed extracts of mango (*Mangifera indica L*.) reduced lipotoxicity induced by high doses of sodium palmitate (PA) in differentiated 3T3-L1 adipocytes. Mango peel (MPE) and mango seed (MSE) extracts significantly lowered PA-induced fat accumulation by reducing lipid droplet (LDs) and triacylglycerol (TAGs) content in adipocytes. We showed that MPE and MSE activated hormone-sensitive lipase, the key enzyme of TAG degradation. In addition, mango extracts down-regulated the adipogenic transcription factor PPARγ as well as activated AMPK with the consequent inhibition of acetyl-CoA-carboxylase (ACC). Notably, PA increased endoplasmic reticulum (ER) stress markers GRP78, PERK and CHOP, as well as enhanced the reactive oxygen species (ROS) content in adipocytes. These effects were accompanied by a reduction in cell viability and the induction of apoptosis. Interestingly, MPE and MSE counteracted PA-induced lipotoxicity by reducing ER stress markers and ROS production. In addition, MPE and MSE increased the level of the anti-oxidant transcription factor Nrf2 and its targets MnSOD and HO-1. Collectively, these results suggest that the intake of mango extract-enriched foods in association with a correct lifestyle could exert beneficial effects to counteract obesity.

## 1. Introduction

Obesity is a multifactorial disease characterized by the accumulation of body fat resulting from excessive food intake, reduced physical activity, environmental factors and genetic susceptibility [1,2]. For decades now, the incidence of obesity has increased in developing countries, representing a public health problem [1]. Hypertrophic expansion of white adipose tissue (WAT) represents an important risk factor for the development of several chronic diseases, including insulin resistance, type II diabetes, non-alcoholic fatty liver disease (NAFLD), cardiovascular diseases and some forms of cancers, such as pancreatic, colorectal, ovarian, thyroid and breast cancers [3,4,5,6].

Excess dietary fat intake has been associated with overweight and fat deposition in mice and humans and represents a serious health risk [7,8,9]. However, the quality of dietary fats has been shown to induce differential lipid storage. In fact, evidence shows that a high intake of saturated long-chain fatty acids (SLFAs), such as palmitic acid (PA), is associated with obesity [10], while a diet containing monounsaturated (MUFAs) and polyunsaturated fatty acids (PUFA), such as oleic or linoleic acid, or medium-chain fatty acids (MFAs), including caprylic acid, capric acid and lauric acid, may have beneficial effects on body weight and obesity [11]. This can be explained considering that certain fatty acids (FAs) are more likely to be stored in adipose tissue versus being oxidated for energy. In particular, SLFAs have lower oxidation rates than MUFAs, PUFAs and MFAs, leading to increased fat storage in white adipose tissue (WAT) [12].

Fat accumulation into adipose tissue due to high consumption of LSFAs produces hypertrophic and dysfunctional adipocytes, leading to a state of chronic low-grade inflammation [13] that contributes to the development of obesity-related diseases [14]. PA induces hypertrophy by increasing lipids droplet (LDs) content, and causes DNA damage in adipocytes in vitro [15]. Moreover, high consumption of PA increases the expression of pro-inflammatory cytokines (TNFα, IL-6, IL-1β) in adipose tissue [16]. The mechanisms through which high levels of LSFAs induce adipocyte disfunction and inflammation in WAT are different. When a large amount of PA is present, adipocytes metabolize it into lysophosphatidylcholine, diacylglicerol (DAG) and ceramides [17]. These compounds have been shown to induce PKC activation, endoplasmic reticulum (ER) stress induction and NF-kB activation [18,19,20].

Several studies suggest that increased oxidative stress is positively correlated with obesity [21]. Obese patients exhibit an abnormal oxidant/antioxidant status with higher levels of oxidative stress markers such as hydroperoxides and carbonyl proteins, while their antioxidant defenses are lower than those of their normal-weight counterparts [22]. The increased presence of reactive oxygen species (ROS) causes extensive oxidative damage to proteins, lipids and DNA, promoting metabolic dysfunction and lipotoxicity in adipocytes [23]. High-fat diets promote oxidative stress in adipose tissue [24]. It has been shown that PA increases ROS production in adipocytes by increasing NADPH oxidase 4 (NOX4) activity [23,24,25]. Moreover, it has been suggested that the elevated bioavailability of FAs can overwhelm the mitochondrial respiratory chain and oxygen consumption, leading to mitochondrial dysfunction and ROS production [26]. Interestingly, oxidative stress and inflammation appear to be closely interlinked in obesity. ROS may activate redox-sensitive transcription factors, such as NF-κB, that transactivate pro-inflammatory cytokines, such as IL-6 and TNF-α [27]. These, in turn, may further induce ROS production, generating a vicious circle between inflammation and oxidative stress [27].

Several studies showed that caloric restriction or increased physical activity lowered fat mass with a consequent reduction of oxidative stress and inflammation-associated obesity [28,29]. In addition, there is an increasing interest in natural antioxidant compounds, such as polyphenols contained in plants, due to their effectiveness against obesity and the related chronic diseases [30,31].

Mango (*Mangifera indica* L.) is a tropical plant belonging to the *Anacardiaceae* family whose cultivation has recently spread to the coastal areas of Sicily (Italy), where the favorable climatic conditions stimulate the growth of the plant and the ripening of the fruit [32]. Mango fruit is appreciated for its nutritive and nutraceutical properties [32,33]. It has been shown that different parts of the plant and of the fruit exert anti-inflammatory, anti-oxidant and anti-tumor effects in in vitro as well as in vivo models because of the presence of a wide range of polyphenols [34,35,36].

In addition, several studies demonstrated that mango also exerts anti-obesity and antidiabetic effects. *Mangifera indica* L. leaf extracts have been shown to reduce adipogenesis in 3T3-L1 adipocytes by decreasing the expression of genes involved in lipid metabolism [37]. In addition, mango juice intake decreases adiposity and inflammation in high-fat-diet-fed obese rats [38], while mango fruit powder reduces insulin resistance and steatosis [39]. Furthermore, it has been shown that fresh mango consumption improves postprandial glucose and insulin responses in obese adults [40]. Arshad et al. demonstrated that the consumption of mango peel powder reduced oxidative stress and dyslipidemia in obese subjects [41]. These studies highlighted the potential of mango as a functional food for the treatment of obesity and related diseases.

The edible part of mango is only the pulp. However, it has been reported that mango peel and seed, which are the main bio-wastes of mango processing, contain high levels of bioactive compounds [42,43]. We previously demonstrated that extracts of mango peel and seed cultivated in Sicily exert anti-adipogenic effects by reducing the differentiation of 3T3-L1 fibroblasts into adipocytes. These effects results from the down-regulation of adipogenic factors such as PPARγ and SREBP as well as the activation of AMPK [43].

Keeping in view the potent health benefits of these mango extracts, the present study was designed to evaluate the ability of mango peel extracts (MPE) and mango seed extracts (MSE) to counteract lipotoxicity induced in adipocytes by SLFAs. To this end, we used an in vitro model in which mature 3T3-L1 adipocytes were treated with high doses of PA, resulting in artificially hypertrophied mature adipocytes. In our model, we examined the effect of mango extracts on PA-induced hypertrophy and oxidative stress. Our data provide evidence that MPE and MSE reduced lipid accumulation and exerted anti-oxidant effects by reducing lipogenesis, inducing lipolysis and counteracting ER stress and ROS increase. The activation of the AMPK and Nrf2 pathways seems to suggest that MPE and MSE reduced lipotoxicity induced by PA in adipocytes.

## 2. Results

### 2.1. MPE and MSE Reduce PA-Induced Toxicity in 3T3-L1 Adipocytes

**Figure 1:**
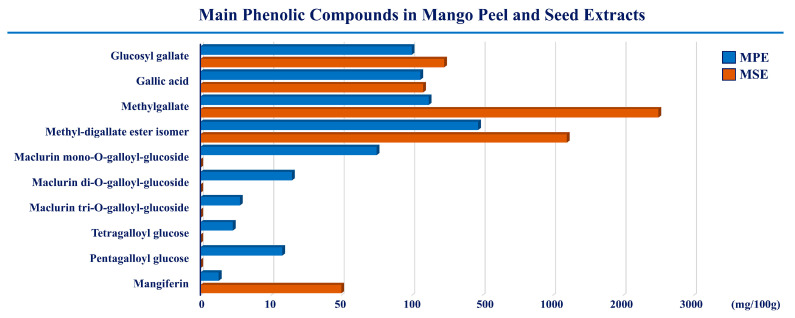
The main phenolic compounds in mango peel and seed extracts. The compositions of MPE and MSE were characterized by HPLC-ESI-MS analysis. Methyl-digallate ester isomer, methyl gallate, gallic acid and glucosyl gallate are the most representative polyphenols in both the extracts.

The present study aimed at investigating whether peel and seed extracts of mango were capable of reducing lipotoxicity exerted by high doses of PA on differentiated 3T3-L1 adipocytes. The compositions of both MPE and MSE have been previously characterized by HPLC-ESI-MS analysis [35,43]. Data showed that both the extracts are rich in polyphenols with antioxidant properties [35,43]. In particular, methyl digallate, methyl gallate, gallic acid and glucosyl gallate were the main phenolic compounds. A representative picture of the main phenolic compounds of MPE and MSE is shown in Figure 1. Moreover, our previous studies demonstrated that 100 μg/mL of MPE or MSE counteracted the adipocyte differentiation of 3T3-L1 cells [43].

**Figure 2:**
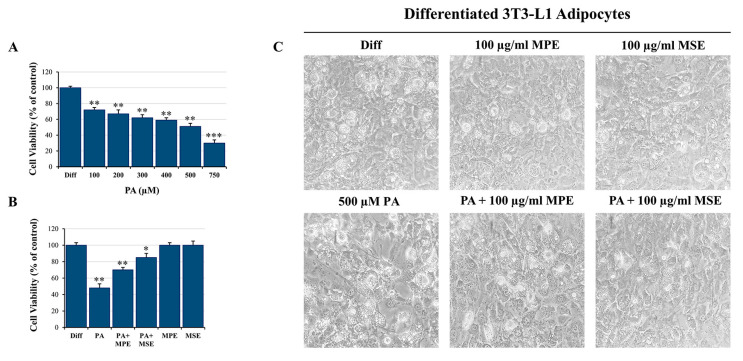
MPE and MSE counteract PA-induced toxicity in 3T3-L1 adipocytes. Differentiated 3T3-L1 adipocytes were exposed for 48 h to different doses of PA alone or in the presence of 100 μg/mL MPE and MSE. (**A**) MTT assays showing the reduction of cell viability induced in differentiated 3T3-L1 adipocytes by different doses of PA. (**B**) MTT assays showing the ability of 100 µg/mL of MPE or MSE to counteract the cytotoxic effect of 500 µM PA in differentiated 3T3-L1 adipocytes. (**C**) Representative phase contrast microscopy images showing the morphological changes induced by 500 µM PA alone or in the presence of 100 µg/mL MPE or MSE in differentiated 3T3-L1 adipocytes. (**A**,**B**) The values reported are the mean ± SD of three independent experiments. The statistical differences between groups were evaluated using a one-way ANOVA test. * *p* < 0.05, ** *p* < 0.01 and *** *p* < 0.001 were significant with respect to differentiated 3T3-L1 adipocytes treated with only vehicle BSA.

In this study, we used an in vitro model in which differentiated 3T3-L1 adipocytes were treated with high doses of PA to generate artificially hypertrophied mature adipocytes [44]. Firstly, 3T3-L1 pre-adipocyte cells were differentiated into adipocytes as reported in Section 4 and then treated for 48 h with different doses of PA (100–750 μM) to evaluate their effect on cell viability, in accordance with other authors [45]. Data obtained by MTT assay demonstrated that PA inhibited cell survival in a dose-dependent manner with a reduction of cell viability of 50% with 500 µM PA (Figure 2A). Notably, the addition of 100 µg/mL MPE or MSE increased cell viability by 46% and 77%, respectively, in comparison with PA-treated adipocytes (Figure 2B). Microscope images highlighted that the number of cells was reduced in PA-treated adipocyte cells with respect to adipocytes co-treated with PA and MPE or MSE (Figure 2C). In addition, signs of toxicity were observed after PA treatment alone that disappeared after the addition of mango extracts (Figure 2C). Thus, in the following experiments, 100 µg/mL MPE or MSE was used to investigate the mechanism underlying the protective effects of mango extracts on lipotoxicity induced by 500 μM PA.

### 2.2. MPE and MSE Reduce Lipid Accumulation in Adipocytes Exposed to High Doses of PA

**Figure 3:**
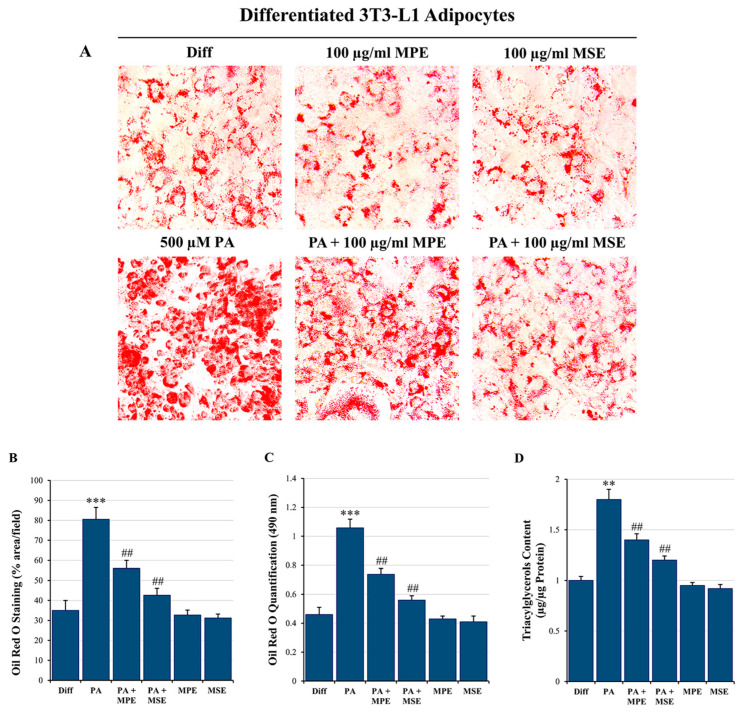
MPE and MSE reduce 3T3-L1 adipocyte hypertrophy induced by high concentrations of PA. 3T3-L1 differentiated adipocytes were treated for 48 h with 500 µM PA alone or in the presence of 100 µg/mL MPE or MSE. (**A**) Representative Oil red O staining microscopy images showing the increase in LDs after treatment with 500 µM PA alone and their reduction when 100 μg/mL MPE or MSE was added (200× original magnification). (**B**) LD content was ascertained by analyzing the percentage area of Oil Red O stained by ImageJ. (**C**) Quantitative Oil red O staining was measured by a spectrophotometer at 490 nm. (**D**) Cellular TAG content was quantified by spectrophotometer at 546 nm. The results are the mean of three independent experiments ± SD. The statistical differences between groups were evaluated using a one-way ANOVA test. ** *p* < 0.01 and *** *p* < 0.001 were significant with respect to differentiated 3T3-L1 adipocytes and ## *p* < 0.01 with respect to PA-treated 3T3-L1 adipocytes.

Excessive lipid availability has been related to adipose tissue hypertrophy [46]. To examine the anti-lipogenic effect of MPE and MSE, differentiated 3T3-L1 adipocytes were treated for 48 h with 500 µM PA in the absence or presence of 100 µg/mL MPE or MSE. Microscope images highlighted that the treatment of mature 3T3-L1 adipocytes with PA increased the content of lipids, as demonstrated by the presence of larger lipid vacuoles with respect to differentiated control 3T3-L1 adipocytes (Figure 2C). Notably, the content of these vacuoles was markedly reduced by MPE and MSE (Figure 2C). These observations were confirmed by staining the cells with Oil Red O (Figure 3A). In comparison with differentiated control adipocytes, 48 h treatment with 500 μM PA resulted in an increase in lipid droplets (LDs) in adipocytes. The addition of 100 µg/mL MPE or MSE to PA-treated adipocytes lowered lipid accumulation in comparison with PA-treated adipocytes. A modest reduction of LDs was also observed in adipocytes not exposed to PA and treated with the extracts alone (Figure 3A). These data were confirmed by microscopic quantification of the Oil Red O staining area (Figure 3B) as well as by measuring the absorbance of the solubilized Oil Red O at 490 nm (Figure 3C). As shown in Figure 3B and 3C, the addition of 100 μg/mL MPE or MSE to PA-treated adipocytes reduced both the staining area and the absorbance of the stained cells by about 30% and 47%, respectively in comparison with PA-treated adipocytes alone. Such a reduction in lipid accumulation was also sustained by measuring the TAG content (Figure 3D). The results showed that the intracellular TAG accumulation increased in PA-treated cells by 80% with respect to untreated differentiated adipocytes. Interestingly, the addition of MPE or MSE to PA-treated cells significantly decreased the TAG content by 23% and 34%, respectively compared with PA-treated adipocytes (Figure 3D).

### 2.3. MPE and MSE Inhibit PPARγ and Activate AMPK

**Figure 4:**
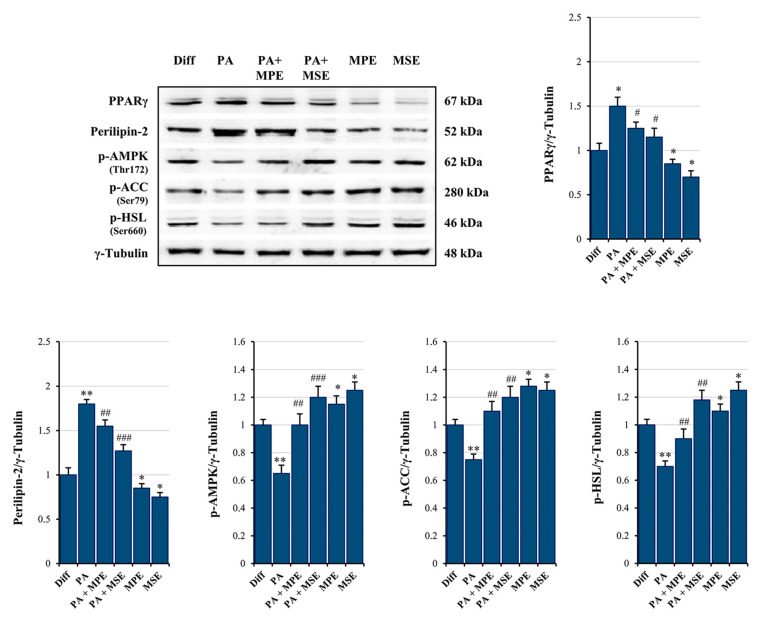
MPE and MSE reduce lipid accumulation, preventing lipogenesis and promoting lipolysis. Differentiated 3T3-L1 adipocytes were treated with 500 µM PA for 48 h in the presence or absence of 100 µg/mL MPE or MSE, as reported in Section 4. Then, cell lysates were analyzed by Western blotting using specific primary antibodies directed against PPARγ, Perilipin-2, phosphorylated AMPK (p-AMPK), phosphorylated ACC (p-ACC) and phosphorylated HSL (p-HSL). Equal amounts of proteins were loaded in each lane (30 μg) as normalized by γ-Tubulin detection. The bar graphs represent the means of three independent experiments ±SD. The statistical differences between groups were evaluated using a one-way ANOVA test. * *p* < 0.05, ** *p* < 0.01 were significant with respect to differentiated 3T3-L1 adipocytes. # *p* < 0.05, ## *p* < 0.01, ### *p* < 0.001 were significant with respect to PA-treated differentiated 3T3-L1 adipocytes.

To investigate the molecular basis for the anti-obesity effect of MPE and MSE, we first evaluated whether mango extracts are capable of reducing the level of PPARγ, the master regulator of adipogenesis [47]. Our data supported the conclusion that PPARγ signaling sustained PA-induced hypertrophy in adipocytes. In fact, we observed an increase of 50% of PPARγ levels in adipocytes treated for 48 h with 500 µM PA, with respect to untreated adipocytes (Figure 4). A concomitant increase in the perilipin-2 levels (80%), a lipid droplet coating protein [48], was observed in PA-treated adipocytes (Figure 4). Notably, the addition of MPE or MSE to PA-treated adipocytes reduced the increase in PPARγ to only 18% and 23%, respectively as well as that in perilipin-2 to only 15% and 30%, respectively, in comparison with PA-treated adipocytes (Figure 4).

Next, we examined whether MPE and MSE affects AMPK activation, a kinase promoting catabolic pathways in adipocytes [49]. As shown in Figure 4, the expression of the phosphorylated and active form of AMPK lowered in PA-treated differentiated 3T3-L1 adipocytes compared with control adipocytes. Interestingly, MPE or MSE alone and in the presence of PA significantly enhanced the phosphorylated form of AMPK (p-AMPK) (Figure 4). This is in line with our previous study demonstrating that MPE and MSE activate AMPK during adipocyte differentiation [43]. Moreover, the addition of MPE or MSE in control adipocytes as well as in PA-treated adipocytes increased the levels of the phosphorylated and inactive form of acetyl-CoA-carboxylase (p-ACC) (Figure 4), the key enzyme of fatty acid synthesis, which is inactivated by phosphorylation by AMPK [49].

Finally, our data also demonstrated that MPE and MSE markedly increased the phosphorylated and active form of hormone sensitive lipase (p-HSL), the enzyme activating lipolysis in adipocytes [50], by 30% and 65%, respectively (Figure 4).

### 2.4. MPE and MSE Reduce PA-Induced ER Stress in 3T3-L1 Adipocytes

**Figure 5:**
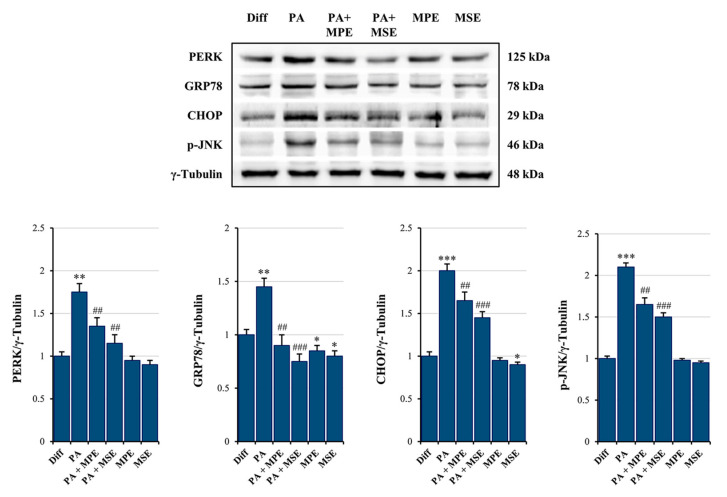
MPE and MSE reduce PA-induced ER stress in 3T3-L1 adipocytes. Differentiated 3T3-L1 adipocytes were treated with 500 µM PA for 48 h in the presence or absence of 100 µg/mL MPE or MSE. Cell lysates underwent Western blotting analysis for ER stress protein markers PERK, GRP78 and CHOP, as well as for phosphorylated JNK (p-JNK). Equal loading of protein (30 µg) was verified by immunoblotting for γ-Tubulin. The bar graphs represent the means of three independent experiments ± SD. The statistical differences between groups were evaluated using a one-way ANOVA test. * *p* < 0.05, ** *p* < 0.01 and *** *p* < 0.001 were significant with respect to differentiated 3T3-L1 adipocytes. ## *p* < 0.01, ### *p* < 0.001 were significant with respect to PA-treated differentiated 3T3-L1 adipocytes.

Elevated levels of FAs, in particular saturated fatty acids (SFAs) such as PA, have been shown to produce ER stress in a number of cell types, including adipocytes [51]. The activation of ER stress, in turn, represents a potential molecular mechanism of lipotoxicity [52]. We thus examined whether high doses of PA induce ER stress in mature adipocytes and the ability of MPE and MSE to counteract it. Interestingly, we observed an increase in ER stress protein markers, evidenced by an up-regulation in the expression of PERK, GRP78 and CHOP, as well as in JNK phosphorylation following the treatment of mature 3T3-L1 adipocytes with 500 µM PA for 48 h (Figure 5). These results suggest that the ER-associated unfolded protein response (UPR) pathway is activated by PA [53]. Notably, the addition of 100 µg/mL MPE or MSE to PA-treated differentiated adipocytes reduced the levels of all ER stress protein markers (Figure 5), thus suggesting the ability of mango extracts to counteract ER stress.

### 2.5. MPE and MSE Prevent PA-Induced ROS Production

**Figure 6:**
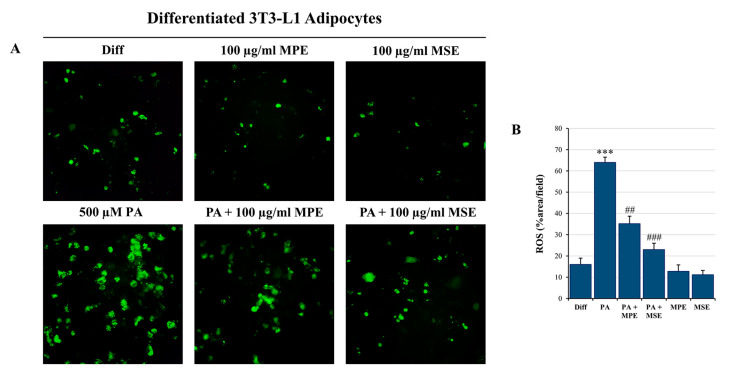
MPE and MSE reduce PA-induced oxidative stress in 3T3-L1 adipocytes, reducing ROS production. Intracellular ROS detection was performed by redox-sensitive fluorochrome H_2_DCFDA. Differentiated 3T3-L1 adipocytes were treated with 500 µM PA for 48 h in the presence or absence of 100 µg/mL MPE or MSE, as reported in Section 4. Then, cells were incubated with 10 µM H_2_DCFDA solution for 30 min at 37 °C. The oxidation of the fluorochrome-generated green fluorescence was visualized by a Leica microscope equipped with a DC300F camera using a FITC filter. (**A**) Representative images of fluorescence microscopy were taken at 200× magnification. (**B**) ROS content was ascertained by analyzing the percentage area with Image J. *** *p* < 0.001 was significant with respect to differentiated 3T3-L1 adipocytes, and ## *p* < 0.01, ### *p* < 0.001 were significant with respect to PA-treated 3T3-L1 adipocytes.

It has been reported that free FAs generate ROS in different cell types, including adipocytes [19,54]. Thus, to evaluate whether PA increased intracellular ROS production, differentiated 3T3-L1 adipocytes were incubated with H_2_DCFDA, a specific fluorescent probe that visualizes intracellular ROS [55]. H_2_DCFDA-associated fluorescence was elevated by 65% after incubation with 500 µM PA for 48 h compared with untreated differentiated 3T3-L1 adipocytes (Figure 6A,B). Interestingly, the addition of 100 µg/mL MPE or MSE markedly reduced ROS content to 35% and 23% compared with adipocytes only treated with PA (Figure 6A,B), thus highlighting that mango extracts counteract ROS production and oxidative stress induced in adipocytes after PA treatment.

**Figure 7:**
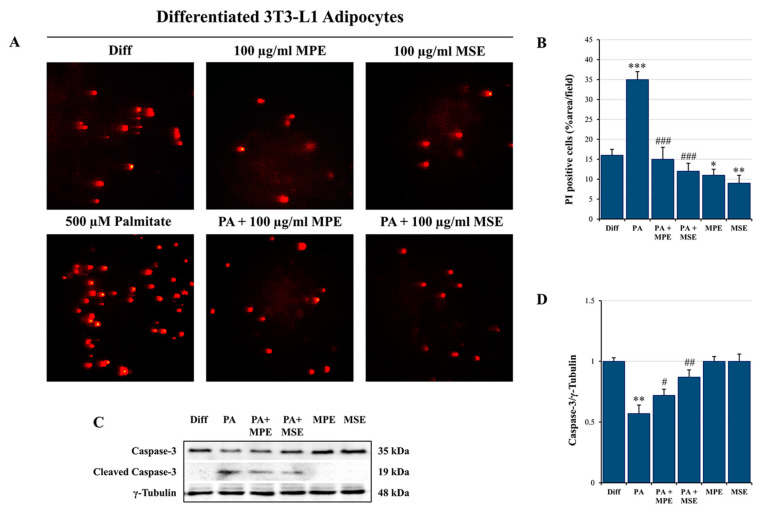
MPE and MSE reduce the cytotoxic effects of PA in 3T3-L1 adipocytes. Propidium iodide (PI) staining of differentiated 3T3-L1 adipocytes treated with 500 µM PA for 48 h in the presence or absence of 100 µg/mL MPE or MSE. (**A**) Representative fluorescence microscopy images were taken at 200× magnification by a Leica microscope equipped with a DC300F camera using a PE filter. (**B**) PI content was ascertained by analyzing the percentage area with Image J. (**C**) Western blotting analysis of the procaspase-3 levels. An equal loading of protein (30 µg) was verified by immunoblotting for γ-Tubulin (**D**). The bar graphs represent the means of three independent experiments ± SD. * *p* < 0.05, ** *p* < 0.01 and *** *p* < 0.001 with respect to differentiated 3T3-L1 adipocytes. # *p* < 0.05, ## *p* < 0.01, ### *p* < 0.001 were significant with respect to PA-treated 3T3-L1 adipocytes.

In addition, propidium iodide (PI) staining of cells confirmed the induction of cytotoxic effects in PA-treated differentiated adipocytes. PA treatment increased cell death by about 35% compared with control adipocyte cells (Figure 7A,B). These effects were counteracted by the addition of 100 µg/mL MPE or MSE that markedly reduced cell death by about 57% and 65%, respectively, with respect to PA-treated adipocytes.

**Figure 8:**
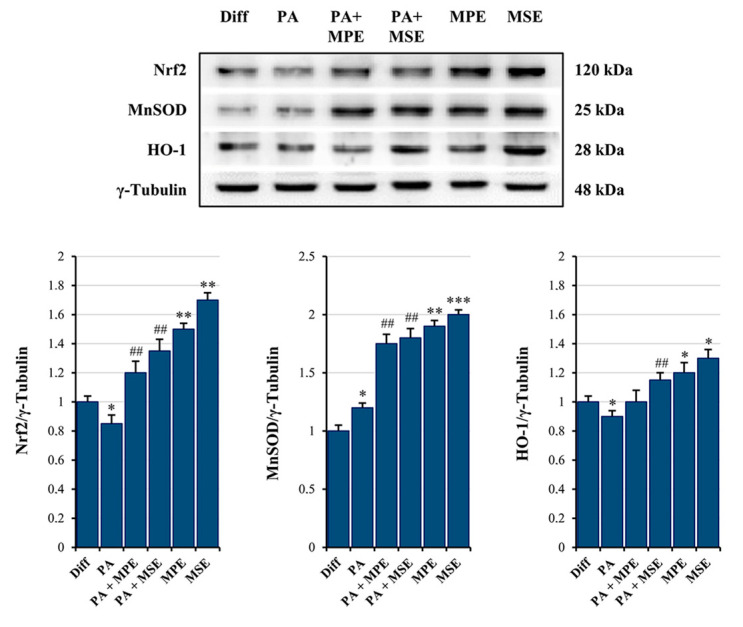
MPE and MSE increase the expression levels of the anti-oxidant molecules. Differentiated 3T3-L1 adipocytes were treated with 500 µM PA for 48 h in the presence or absence of 100 µg/mL MPE or MSE. Cell lysates underwent Western blotting analysis for Nrf2, MnSOD and HO-1. An equal loading (30 µg) of proteins was verified by immunoblotting for γ-Tubulin. The bar graphs represent the means of three independent experiments ±SD. The statistical differences between groups were evaluated using a one-way ANOVA test. * *p* < 0.05, ** *p* < 0.01 and *** *p* < 0.001 were significant with respect to differentiated 3T3-L1 adipocytes. ## *p* < 0.01 was significant with respect to PA-treated differentiated 3T3-L1 adipocytes.

The cytotoxic effects induced by PA in adipocytes seem to be related to apoptosis induction. Pro-caspase-3 is a master apoptosis protein marker cleaved in active form during this process [56]. PA treatment decreased the level of pro-caspase-3 by 43% (Figure 7C,D) and promoted the appearance of the cleaved active form of caspase-3. Notably, caspase activation was counteracted by the addition of MPE or MSE (Figure 7C,D). Our previous studies provided evidence that MPE and MSE contain factors capable of exerting ROS scavenger effects during 3T3-L1 adipocyte differentiation [43]. These effects have been correlated with the ability of mango extracts to increase Nrf2, the main antioxidant transcription factor [57], during adipocyte differentiation [43]. In accordance with our previous data, we demonstrated that in PA-treated adipocytes, MPE or MSE increased the level of Nrf2 by about 40% and 60%, respectively (Figure 8). Our data also showed that the levels of MnSOD and HO-1, two scavenger enzymes transcriptionally regulated by Nrf2 [57,58], markedly increased after treatment with MPE or MSE. In particular, the increase in MnSOD in the presence of MPE or MSE was estimated to be 46% and 50%, while that of HO-1 was estimated to be 12% and 28%, respectively.

## 3. Discussion

The current study was designed to investigate whether extracts of mango peel (MPE) and seed (MSE) could ameliorate PA-induced lipotoxicity in adipocytes. Peel and seed are the main bio-waste products of mango processing. In an earlier study, we demonstrated that MPE and MSE have the ability to reduce the number of adipocytes by preventing adipocyte differentiation of 3T3-L1 pre-adipocyte cells [43]. In the present study, we provided evidence that MPE and MSE are also capable of lowering adipocyte hypertrophy induced by high doses of PA, the main saturated long fatty acid present in the diet [59]. Notably, we demonstrated that MPE and MSE reduced PA-induced fat accumulation, as evidenced by the decrease in LD and TAG content in differentiated 3T3-L1 adipocytes co-treated with PA and MPE or MSE.

The ability of MPE and MSE to reduce lipid content in PA-treated adipocytes results from both stimulation of lipolysis and inhibition of lipogenesis. PPARγ is a transcription factor that has been reported to play a critical role in adipocyte hypertrophy under high fat diets [60]. We provided evidence that the PPARγ level increased under PA-treatment in differentiated 3T3-L1 adipocytes. Notably, this effect was markedly counteracted by the addition of MPE or MSE to PA-treated adipocytes. These results are in line with our previous data demonstrating that MPE and MSE counteract 3T3-L1 adipocyte differentiation by reducing the level of PPARγ and its target FABP4 [43].

Furthermore, our data showed that MPE and MSE significantly enhanced the phosphorylation of AMPK and its substrate acetil-CoA carboxylase (ACC) in both controls and PA-treated adipocytes, thus suggesting a role of AMPK activation in reducing lipogenesis induced by MPE and MSE. AMPK is an important regulator of lipid metabolism [61]. Activation of AMPK by phosphorylation increases lipolysis and fatty acid oxidation, while inhibiting lipogenesis [62]. AMPK inactivates by phosphorylation ACC, the key enzyme of fatty acid synthesis [63], leading to the reduction of fatty acid synthesis [64]. Different phenolic compounds contained in plants and fruits, such as quercetin, curcumin, resveratrol and gallic acid, exert anti-obesity effects by activating AMPK [61]. We previously characterized the composition of peel and seed extracts of Sicilian mango fruits by HPLC/MS and demonstrated the presence of different polyphenols, among which methyl digallate and methyl gallate are the most represented components [34,43]. These compounds could be responsible for the anti-lipogenic effects of the mango extracts. In line with this conclusion, Fang et al. [65] demonstrated that gallotannin derivatives from mango counteract adipogenesis by activating AMPK. In addition, Lu, et al. showed that gallic acid reduced lipogenesis and improved liver steatosis by activating AMPK [66]. This effect could result by a direct interaction of gallic acid with AMPKα/β subunits, as evidenced by computational docking analysis [66]. Finally, mangiferin, a polyphenol derived from *Mangifera indica* promotes browning of adipocytes by activating AMPK [67].

In this study, we also provided evidence that MPE and MSE increased the level of the phosphorylated and active forms of hormone-sensitive lipase (HSL), the key lipase activating lipolysis of TAGs in adipocytes, in PA-treated adipocytes [68]. Different lipolytic agents activate HSL by increasing cAMP levels, with the consequent activation of cAMP-dependent protein kinase (protein kinase A; PKA). This enzyme in turn phosphorylates and activates HSL [69]. MSE and MPE could activate HSL because of their content of polyphenols. In line of this conclusion, it has been shown that different polyphenols are able to increase cAMP by inhibiting phosphodiesterase, the enzyme that degrades cAMP [70,71].

A high content of SLFAs has been associated with lipotoxicity in adipocytes as a consequence of ER stress induction [72]. Notably, when present at a high level, PA is metabolized into saturated DAG and saturated lysophosphatidylcholine [19]. These PA-derived metabolites accumulate in the ER, causing destructive changes in its structure and the activation of ER stress sensors [19]. In line with these observations, we demonstrated that PA treatment enhanced the ER stress markers GRP78, PERK and CHOP as well as activated JNK by increasing its phosphorylated form in differentiated 3T3-L1 adipocytes. ER stress is a protective cellular mechanism that initiates the unfolded protein response (UPR) to restore cellular homeostasis [73]; however, in severe ER stress, the adaptive response fails and apoptotic cell death is induced [73]. In obese animals, elevated ER stress is present in different organs [74,75]. In this condition, ER stress-induced UPR activates JNK, which in turn promotes apoptosis by inhibiting the mitochondrial respiratory chain and activating caspases [76]. Our data confirmed that PA causes lipotoxicity in differentiated adipocytes, as evidenced by cell viability reduction, increased PI-positive cells and caspase-3 activation. Interestingly, MPE and MSE counteracted PA-induced ER stress by lowering all ER stress markers, GRP78, PERK and CHOP, as well as p-JNK. Concomitantly, mango extracts restored cell viability, reduced PI-positive cells and the activation of caspase-3 induced by PA treatment, thus suggesting their protective effects against lipotoxicity induced by high levels of SFAs in adipocytes. Furthermore, we demonstrated that PA treatment increased in 3T3-L1 adipocytes the level of ROS, as evidenced by staining adipocytes with H_2_DCFDA. This finding is in line with previous reports demonstrating that high levels of fatty acid increase oxidative stress in adipocytes [77]. It has been reported that ceramide and DAG, which are fatty acid-derived lipid metabolites, activate NADPH oxidase (NOX), enhancing the ROS level in adipocytes [78]. In addition, dysfunction of the mitochondrial respiratory chain in obesity can amplify oxidative stress and inflammation [79]. ROS production has been shown to activate JNK, which mediates activation of NF-κB and AP-1 [80] with the consequent enhanced expression of pro-inflammatory cytokines, such as IL-6 and TNFα. Notably, we showed that the production of ROS in PA-treated adipocytes was markedly reduced by the addition of MPE and MSE. This effect could be a consequence of the high content of polyphenols in mango extracts. This is in line with the observation that methyl-gallate, the main component of MPE and MSE, protects the cells against oxidative damage through its ROS scavenger ability [81]. Furthermore, the lowering in ROS content induced by MPE and MSE could be a consequence of the up-regulation of Nrf2 and its transcriptional targets MnSOD and HO-1, two important antioxidant enzymes [34,43]. The activation of Nrf2, the main transcriptional factor against exogenous and endogenous oxidative stress injury [82,83], has been reported in different dietary polyphenols, including resveratrol, gallic acid and caffeic acid [84]. The mechanisms underlying Nrf2 activation include increased Nrf2 nuclear translocation, inhibition of Keap1-Nrf2 interaction and enhanced Keap1 ubiquitination [84]. Finally, MPE and MSE could reduce ROS levels and oxidative stress in adipocytes by activating AMPK. In line with this hypothesis, the deregulated activity of AMPK has been associated with an inflammatory state in in vivo models of obesity and obese patients [85]. Indeed, the activation of AMPK signaling has been shown to protect against oxidative stress by suppressing NOX [86] and mitochondrial dysfunction [87].

## 4. Materials and Methods

### 4.1. MPE and MSE Preparation

Peel and seed extracts were obtained from mango fruits (*Mangifera Indica* L.) cultivated in Sicily (Italy), as reported before [43]. In particular, after washing with distilled water, the peels and seeds of mango fruits were cut and lyophilized (Hetosicc Lyophilizer Heto CD 52-1). Then, an ethanol:PBS 1:1 solution was used in order to solubilize the lyophilized products by keeping them overnight at 37 °C under constant shaking. The final concentration of both the extracts was 75 mg/mL. Then, we centrifuged both the extracts of MPE and MSE at 120× g for 10 min. The obtained supernatants of MPE and MSE were centrifuged again at 15,500× g for 10 min and then the extracts (supernatants) were frozen at −20 °C until use. MPE and MSE working solutions were prepared by diluting them to the final concentration in culture medium. The final concentration of ethanol in the extracts showed no toxicity on differentiated 3T3-L1 adipocytes.

### 4.2. PA Solution Preparation

PA was solubilized in an EtOH 10% solution (25mM) in a heated and stirred water bath at 65 °C for 15 min. Once completely solubilized, a 500 µM working dilution was appropriately prepared in culture medium containing 5% BSA and incubated at 37 °C for 1 h under constant shaking to ensure their conjugation before adding it to differentiated 3T3-L1 adipocytes. Vehicle containing 5% BSA was used as control (differentiated 3T3-L1 adipocytes, Diff).

### 4.3. Cell Cultures

A mouse 3T3-L1 pre-adipocyte cell line from the American Type Culture Collection (ATCC) was maintained in culture as monolayer in flasks of 75 cm^2^, at 37 °C and in a 5% CO_2_ humidified incubator in DMEM (Euroclone, Pero, Italy), supplemented with 10% (*v*/*v*) heat-inactivated fetal bovine serum (FBS; Euroclone, Pero, Italy), 2 mM L-glutamine (BioWest SAS, Nuaillé, France), 100 U/mL penicillin and 50 µg/mL streptomycin (Euroclone, Pero, Italy). Once 80% of confluence was reached, 3T3-L1 pre-adipocytes were detached from the flasks using trypsin-EDTA (0.5 mg/mL trypsin and 0.2 mg/mL EDTA) and seeded according to the experimental conditions. All compounds and reagents used for our experiments, unless otherwise stated, were purchased from Sigma-Aldrich (Milan, Italy).

### 4.4. Adipocyte Differentiation, Reagents and Treatments

Differentiated 3T3-L1 adipocytes were obtained from 3T3-L1 pre-adipocyte cells (undifferentiated cells) as previously reported [43]. In particular, 3T3-L1 cells were seeded at 0.2 × 10^5^/well in 24-well plates or 0.8 × 10^5^/well in 6-well plates and kept until the confluence was reached. Then, after two days post-confluence, undifferentiated cells were incubated with a differentiation culture medium constituted by DMEM supplemented with 10% (*v*/*v*) heat-inactivated FBS, 2 mM L-glutamine, 1% Non-Essential Amino Acids, 100 U/mL penicillin and 50 µg/mL streptomycin, containing the pro-differentiative agents 0.5 mM 3-isobutyl-1-methylxanthine (IBMX), 1 µM dexamethasone and 10 µg/mL insulin. After another three days, the differentiation medium was removed and maintenance culture medium (DMEM supplemented with 10% (*v*/*v*) heat-inactivated fetal bovine serum, 2 mM L-glutamine, 1% Non-Essential Amino Acids 100 U/mL penicillin and 50 µg/mL streptomycin containing 10 µg/mL insulin) was added and left for 5 days. Complete differentiation was reached at day 8 when the cells showed typical features of mature adipocytes, such as LD formation and TAG accumulation. PA alone or in the presence of 100 μg/mL MPE or MSE was added to differentiated 3T3-L1 adipocytes and kept for 48 h.

### 4.5. Cell Viability Assessment

Cell viability was evaluated by measuring mitochondrial dehydrogenase activity using 3-(4,5-dimethylthiazol-2-yl)-2,5-diphenyltetrazolium bromide (MTT), as reported before [88]. For the cell viability assay, undifferentiated 3T3-L1 cells were seeded in 96-well plates (8 × 10^3^ cells/well) until complete differentiation. Then, differentiated 3T3-L1 adipocyte cells were exposed to different concentrations of PA alone or in the presence of 100 μg/mL MPE or MSE for 48 h. Then, 20 µL of MTT reagent (11 mg/mL diluted in PBS) was added to each well and incubated for another 2 hours at 37 °C. The colored crystals of the formazan produced by viable cells were dissolved by adding 100 μL of lysis buffer containing 20% sodium dodecyl sulphate in 50% N,N-dimethylformamide, pH 4.0 and the absorbance was measured by a microplate reader (OPSYS MR, Dynex Technologies, Chantilly, VA, USA) at 540 nm with a reference wavelength of 630 nm. Cell viability was measured as the percentage of the optical density (OD) values found in treated cells compared with those found in untreated cells as control.

The cytotoxic effects of PA on differentiated 3T3-L1 adipocyte cells were also evaluated by propidium iodide (PI) staining. Differentiated cells were treated with 500 µM PA alone or together with 100 μg/mL MSE or MPE. After 48 h of treatment, cells were washed and stained with PI. After a short incubation at the dark, the fluorochrome in excess was removed and the cells were analyzed by fluorescence microscopy using excitation and emission wavelengths appropriate for PI fluorescence (λex = 488 nm and λem = 610/620nm).

### 4.6. Oil Red O Staining of Treated Mature 3T3-L1 Adipocytes

Oil Red O staining (Sigma-Aldrich, St. Luois, MO, USA) was performed for evaluating LD accumulation. Oil Red O stock solution was prepared by solubilizing 0.35 gr in 100 mL isopropanol 100%. Once differentiated in a 24-well plate, differentiated 3T3-L1 adipocytes were fixed by incubation in 10% formaldehyde for 30 min, washed with PBS and rinsed with 60% isopropanol for 5 min until they were completely dry. Fixed cells were then stained with Oil Red O working solution (3:2, stock solution—dH_2_O) for 10 min and then washed with dH_2_O several times. Red pixel areas, stained by Oil Red O, detecting LDs, were divided by the total area scanned. The whole bottom surface of a single well from a 24-well plate was analyzed for the establishment of LD production. A Leica DM-IRB microscope was used and pictures were taken by a Leica DC300F digital camera with a Leica IM50 software, as representative images of the experimental conditions. The pictures were analyzed in ImageJ, converted into high-contrast black and white images to visualize LDs and scored as the percentage area per field. Finally, Oil Red O quantification was performed by measuring its absorbance at 490 nm after extraction of the dye by 100% isopropanol for 10 min. The percentages of the OD values found in treated cells were compared with those found in untreated differentiated 3T3-L1 cells as control.

### 4.7. ROS Detection

ROS production was detected through the oxidation of the cell-permeant 2′,7′-dichlorodihydrofluorescein diacetate (H_2_DCFDA) (Molecular Probe, Life Technologies, Eugene, OR, USA) dye, as reported before [89]. Differentiated 3T3-L1 adipocytes were treated with 500 μM PA in absence or presence of 100 μg/mL MPE or MSE for 48 h. Then, the cells were washed in PBS and incubated with 10 μM H_2_DCFDA dye for 30 min in the dark and in the presence of 5% CO_2_ at 37 °C. At the end of incubation, the fluorochrome in excess was removed washing in PBS and the fluorescent 2′,7′-dichlorofluorescein (DCF), produced by intracellular oxidation, was analyzed by fluorescence microscopy using excitation and emission wavelengths appropriate for green fluorescence (FITC filter with λex = 485 nm and λem = 530 nm).

### 4.8. TAGs Evaluation

Differentiated 3T3-L1 adipocytes were treated with 500 μM PA in absence or presence of 100 μg/mL MPE or MSE for 48 h. Then, the cells were lysed with 5% NP-40 and the number of TAGs in the supernatants was quantified by a spectrophotometric commercial kit for triglyceride determination (SENTINEL C H. SpA, Milan, Italy) [43]. A standard curve with different TAG concentrations, normalized to total cellular protein content measured by Bradford assay, was used for quantifying the samples’ TGA concentrations.

### 4.9. Western Blot Procedures

Protein levels were detected by western blotting analysis. Differentiated and treated cells were lysed as reported before [90]. Bradford Protein Assay was used to quantify protein concentration (Bio-Rad Laboratories S.r.l., Segrate, Milan, Italy). Afterwards, the same number of proteins (30 μg/sample) was loaded and underwent sodium dodecyl sulfate (SDS)-polyacrylamide gel electrophoresis (PAGE). Finally, gels were blotted onto a nitrocellulose membrane (Bio-Rad).

Immunodetection was then performed, incubating the filters with specific primary antibodies against PERK (ab65142) purchased from Abcam (Cambridge, UK), namely, GRP78 (sc-166490), phosphorylated-JNK (sc-6254), CHOP (sc-793), PPARγ (sc-7273), MnSOD (sc-133254) and caspase-3 (sc-65487), all purchased from Santa Cruz Biotechnology (Santa Cruz, CA, USA). Phosphorylated-ACC (#07-303) was purchased from EMD Millipore Corporation (Temecula, 40 CA, USA), phosphorylated-AMPKα (#2535) and phosphorylated-HSL (#4126) were purchased from Cell Signaling (Danvers, MA, USA); Nrf2 (NBP1-32822) and Perilipin-2 (NB110-40877SS) was purchased from Novus Biologicals (Bio-Techne SRL, Milan, Italy); additionally, HO-1, Heme Oxygenase 1 (orb5455) was purchased from Biorbyt Ltd. (Cambridge, UKi). Immunoreactive signals, developed through HPR-conjugated secondary antibodies (Amersham, GE Healthcare Life Science, Milan, Italy), were detected using enhanced chemiluminescence (ECL) reagents (Cyanagen, Bologna, Italy) and obtained with ChemiDoc XRS (Bio-Rad, Hercules, CA, USA).

A quantification of the signal was performed by Quantity One 1-D Analysis software (Bio-Rad) and γ-Tubulin (T3559; Sigma-Aldrich) was used for loading normalization.

### 4.10. Statistical Analysis

All the experiments and their determinations were performed in triplicate. Data were represented as mean ± S.D and the statistical significance was provided. Data analysis was performed using the GraphPadPrismTM 4.0 software (Graph PadPrismTM Software Inc., San Diego, CA, USA). The differences between groups were evaluated using Tukey’s test following one-way ANOVA test. A *p*-value < 0.05 was considered the threshold for statistical significance. When not specified, the data were not significant with respect to the related control.

## 5. Conclusions

**Figure 9:**
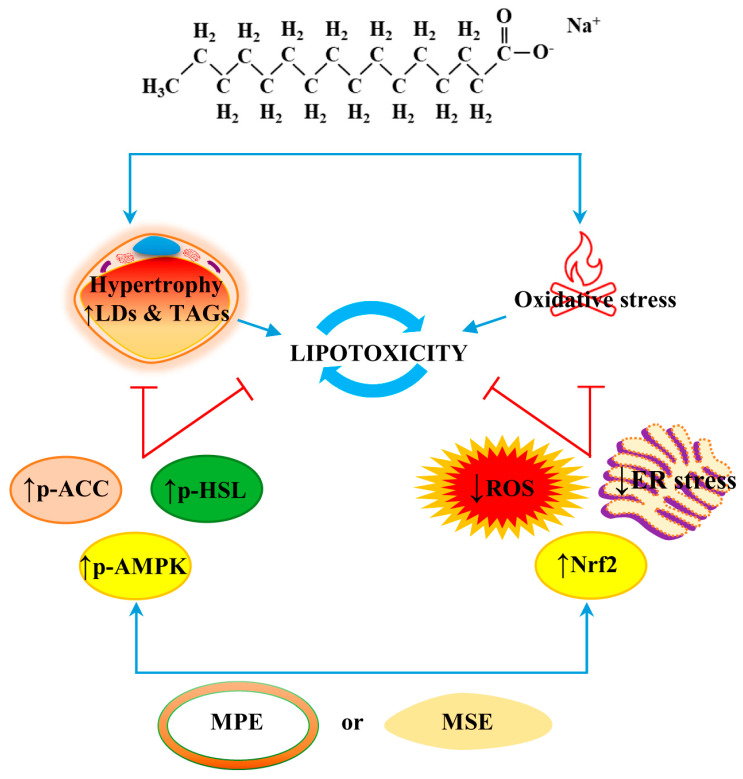
MPE and MSE counteracted lipotoxicity induced by PA in differentiated adipocytes. MPE and MSE lowered fat accumulation induced by high doses of PA in differentiated 3T3-L1 adipocytes, as demonstrated by the reduction of LD and TAG contents. These MPE and MSE anti-lipogenic effects seem to be mediated by the activation of HSL and inhibition of ACC as a result of AMPK activation. MPE and MSE also counteracted PA-induced ER stress and ROS increase in adipocytes. The anti-oxidative effects of MPE and MSE could be ascribed to the activation of the Nrf2/OH-1/MnSOD pathway. Reduced fat content and oxidative stress production could protect the cells from PA-induced cytotoxicity.

In conclusion, the present study demonstrated that MPE and MSE protect against PA-induced lipotoxicity in differentiated 3T3-L1 adipocytes by reducing lipid content and oxidative stress. These anti-obesity effects of MPE and MSE might partly involve the inhibition of lipogenesis, the activation of lipolysis and the induction of antioxidant effects. A representative picture of the anti-lipolytic and anti-oxidative effects of MPE and MSE is reported in Figure 9. In light of the chemical data providing evidence of MSE and MPE composition, we wondered about the putative phytochemicals responsible for the effect observed in 3T3-L1 adipocytes exposed to MPE or MSE treatment. A possible candidate seems to be methyl gallate. This is a phenolic compound that is the most represented phytochemical in our tested mango extracts. Our hypothesis is also sustained by experimental evidence reported by Roh et al. [91] demonstrating that methyl gallate is able to counteract the lipid accumulation in 3T3-L1 cells and could represent a good candidate as an anti-obesity agent. However, we cannot exclude that the ability of MPE and MSE to counteract PA lipotoxicity, and as hypertrophy and ER stress induced by PA exposure could be ascribed to a combined or synergistic effect among the different phytochemicals identified in mango. To better elucidate this aspect, in our future studies we will test mango phytochemicals as compounds alone and their combinations on 3T3-L1 cells.

Our data offer novel perspectives suggesting that MPE and MSE may be associated with the reduced metabolic dysfunction of adipose tissue induced by high levels of SLFAs. Thus, the development of mango extract-rich foods could be useful to counteract obesity and its consequences.

## Data Availability

The data presented in this study are available in this manuscript.
